# Membranes for Energy Conversion

**DOI:** 10.3390/membranes13080735

**Published:** 2023-08-17

**Authors:** V. María Barragán

**Affiliations:** Department of Structure of Matter, Thermal Physics and Electronics, Faculty of Physics, Complutense University of Madrid, Plaza de Ciencias, 1, 28040 Madrid, Spain; vmabarra@ucm.es

In the modern world, the level of global energy consumption continues to increase, with current methods of energy generation still greatly dependent on fossil fuels, which will become less accessible in the not-so-distant future. In addition, the increase in energy prices combined with environmental problems caused by the excessive emission of greenhouse gases have sparked a growing interest in the development of alternative energy sources. In addressing this challenge, membrane technology is a promising alternative for energy conversion; its low environmental impact is rapidly piquing the interest of researchers worldwide. Membranes have the potential to become a key element of the transition to a more energetically sustainable world.

From the perspective of energy conversion, the potential applications of membranes are numerous. They can be used as electrolytes in membrane-based fuel cells and electrolyzers to produce hydrogen; as separators in lithium batteries, producing blue energy via reverse electrodialysis; in thermoelectric and electrokinetic energy conversion, etc. Some membrane technologies are already being applied in industries at a large scale, while others remain in the early stages of development. However, in any case, we continue to face the major challenge of making crucial breakthroughs in membrane science and technology.

This Special Issue, entitled “Membranes for Energy Conversion”, set out with the aim of collating high-quality research on different aspects of the important role of membranes in energy conversion systems. As a result of our proposal, six articles, one communication and one review were published.

Six of these published papers focus on membranes themselves as a key part of energy conversion systems [1,2,3,4,5,6]. The issue of proton-exchange membrane degradation in fuel cells was analyzed by Robert at al. [1]. The authors presented an innovative chemical-mechanic ex situ device to investigate membrane aging and demonstrated that perfluorosulfonic acid membranes were significantly degraded by the impact of conjoined chemical–mechanical stress.

The possibility of using ion-exchange membranes as thermoelectric material was investigated by Barragán [2]. The paper focuses on the experimental relationship between short-circuit current and temperature gradient in a membrane-based thermocell. The results demonstrate the major significance of the membrane system’s properties on the efficiency of the process.

Schiavone et al. [3] focused on proton exchange membranes. These membranes are highly sought after in energy conversion applications. In their paper, a relationship between the morphology of hydrated domains in sulfonated semi-crystalline syndiotactic-polystyrene doped with fullerenes was reported. The results show the improved proton conductivity of these membranes due to the formation of hydrated domains.

In the paper by Nofal et al. [4], new polymer electrolyte membranes were fabricated by doping polyninyl alcohol–methylcellulose with ammonium iodine. Structural and electrical properties of the polymer blend electrolyte were analyzed using different techniques. The results demonstrate that the addition of ammonium iodine increased overall conductivity and that a relatively electrochemically stable electrolyte was obtained, which makes these membranes promising candidates for use in electrochemical energy storage devices.

In both alkaline direct alcohol fuel cells and alkaline alcohol solution electrolysis, anion-exchange membranes are used as electrolytes. In these membrane-based fuel cell technologies, one of the major problems is that alcohol crossover through the membrane strongly affects system performance. Therefore, the design of new membranes with low alcohol diffusion for these applications is vital. In this context, Fernández-Nieto et al. [5] studied the alcohol diffusion of several anion-exchange membranes doped with different alkaline agents and showed that the membrane structure, type of doping, and type of alcohol affected this crucial property.

Limited lithium resources and a predicted sharp increase in prices have caused further research to be conducted on sodium-ion batteries. The research on appropriate materials to use as substitutes for the liquid and gel electrolytes of typical batteries is required, and membranes are shown to be a promising alternative. In another study, Krupina et al. [6] researched polyelectrolytes with unipolar cationic conductivity based on Nafion membranes. The effect of swelling temperature on physicochemical and electrotransport properties was analyzed using different methods. The results demonstrate that it was possible to expand the operating temperature range of a sodium battery as a function of the polymer electrolyte composition and the conditions for its preparation.

Research on proton batteries is currently in its early stages. The device uses the principle of proton exchange membrane water electrolysis for charging, and the proton exchange membrane fuel cell for discharging. The major difference between a proton battery and a proton exchange membrane fuel cell is that hydrogen is stored in the battery as ions. In the study by Lee at al. [7], a flexible five-in-one sensor was developed to be embedded in a proton battery. The objective was to obtain important parameters instantly to more precisely master the battery and to obtain useful information for prolonging battery life and enhancing its performance.

Finally, in their review paper, Kamaroddin et al. [8] focused on using membrane-based electrolysis to obtain hydrogen, a zero-carbon energy source that could be the basis of future energy systems. Various types of water splitting technologies and membranes for electrolyzers are described. This review addresses the challenges, prospects, and future trends of this important field of research and makes critical suggestions regarding the implementation of comprehensive membrane-based electrolytic systems.

In this Special Issue, we have collated important research contributions from different perspectives of membrane applications for energy conversion. These membrane systems constitute a very widespread field of research, one that is impossible to cover in a single Special Issue. The papers featured here illustrate the importance of membranes in energy conversion systems, providing readers with a comprehensive summary and promoting further research in this field.

I express my heartfelt thanks to all authors for their relevant contributions to this Special Issue and for the time and effort made towards conveying their ideas with the utmost clarity, as well as to the reviewers for their effort in reviewing and improving the quality of the submitted papers.

## References

[1] Robert M., El Kaddouri A., Perrin J.-C., Mozet K., Dillet J., Morel J.-Y., Lottin O. (2021). The Impact of Chemical-Mechanical Ex Situ Aging on PFSA Membranes for Fuel Cells. Membranes.

[2] Barragán V.M. (2021). Short-Circuit Current in Polymeric Membrane-Based Thermocells: An Experimental Study. Membranes.

[3] Schiavone M.M., Zhao Y., Iwase H., Arima-Osonoi H., Takata S.-I., Radulescu A. (2022). On the Proton Conduction Pathways in Polyelectrolyte Membranes Based on Syndiotactic-Polystyrene. Membranes.

[4] Nofal M.M., Aziz S.B., Brza M.A., Abdullah S.N., Dannoun E.M.A., Hadi J.M., Murad A.R., Al-Saeedi S.I., Kadir M.F.Z. (2022). Studies of Circuit Design, Structural, Relaxation and Potential Stability of Polymer Blend Electrolyte Membranes Based on PVA:MC Impregnated with NH4I Salt. Membranes.

[5] Fernández-Nieto A., Muñoz S., Barragán V.M. (2022). Alcohol Diffusion in Alkali-Metal-Doped Polymeric Membranes for Using in Alkaline Direct Alcohol Fuel Cells. Membranes.

[6] Krupina A.A., Kayumov R.R., Nechaev G.V., Lapshin A.N., Shmygleva L.V. (2022). Polymer Electrolytes Based on Na-Nafion Plasticized by Binary Mixture of Ethylene Carbonate and Sulfolane. Membranes.

[7] Lee C.-Y., Chen C.-H., Cheong J.-S., Chien Y.-H., Lin Y.-C. (2021). Flexible 5-in-1 Microsensor Embedded in the Proton Battery for Real-Time Microscopic. Membranes.

[8] Kamaroddin M.F.A., Sabli N., Abdullah T.A.T., Siajam S.I., Abdullah L.C., Jalil A.A., Ahmad A. (2021). Membrane-Based Electrolysis for Hydrogen Production: A Review. Membranes.

